# MIT-Skywalker: considerations on the Design of a Body Weight Support System

**DOI:** 10.1186/s12984-017-0302-6

**Published:** 2017-09-06

**Authors:** Rogério Sales Gonçalves, Hermano Igo Krebs

**Affiliations:** 1 Federal University of Uberlândia/Brazil, School of Mechanical Engineering, Av. João Naves de Ávila 2121 Campus Santa Monica CX 593, Uberlândia, MG CEP 38408-100 Brazil; 2 Mechanical Engineering Department, The Eric P. and Evelyn E. Newman Laboratory for Biomechanics and Human Rehabilitation, Massachusetts Institute of Technology – MIT, Boston, USA; 3 Mechanical Engineering Department, Principal Research Scientist & Lecturer MIT, Room 3-137 Massachusetts Institute of Technology, 77 Massachusetts Avenue, Cambridge, MA 02139-4307 USA; 4 Neurology Department, University of Maryland, School of Medicine, Baltimore, USA; 5 Mechanical Science and Bioengineering Department, Osaka University, Suita, Japan; 6 Fujita Health University, School of Medicine, Toyoake, Japan; 7 Newcastle University, Institute of Neuroscience, Newcastle, UK; 8 Loughborough University, The Wolfson School of Engineering, Loughborough, UK

**Keywords:** Rehabilitation robotics, Lower extremity, Gait, Balance, Body weight support system

## Abstract

**Background:**

To provide body weight support during walking and balance training, one can employ two distinct embodiments: support through a harness hanging from an overhead system or support through a saddle/seat type. This paper presents a comparison of these two approaches. Ultimately, this comparison determined our selection of the body weight support system employed in the MIT-Skywalker, a robotic device developed for the rehabilitation/habilitation of gait and balance after a neurological injury.

**Method:**

Here we will summarize our results with eight healthy subjects walking on the treadmill without any support, with 30% unloading supported by a harness hanging from an overhead system, and with a saddle/seat-like support system. We compared the center of mass as well as vertical and mediolateral trunk displacements across different walking speeds and support.

**Results:**

The bicycle/saddle system had the highest values for the mediolateral inclination, while the overhead harness body weight support showed the lowest values at all speeds. The differences were statistically significant.

**Conclusion:**

We selected the bicycle/saddle system for the MIT-Skywalker. It allows faster don-and-doff, better centers the patient to the split treadmill, and allows all forms of training. The overhead harness body weight support might be adequate for rhythmic walking training but limits any potential for balance training.

## Background

Presently an estimated 6.6 million Americans have survived a stroke [1]. Projections from the American Heart Association suggest that this number will be swelled by an additional 3.4 million people by 2030 [1] and the majority of stroke survivors will experience some motor deficits [2, 3]. Although there are a few treatment alternatives to improve cerebral perfusion and neuro-protection after stroke [2, 4], the only way, at this time, for neuro-recovery to ameliorate and reduce the consequences of central nervous system injury is through physical or occupational therapy delivered by clinicians and potentially augmented by robotic tools [4, 5].

Different methods of gait rehabilitation have been proposed over the years and body weight supported treadmill training (BWSTT) emerged as the approach of choice in early 2000 [6–9]. Patients were suspended in a body weight support harness over a treadmill, while two or sometimes three therapists assisted the patient: one or two sitting adjacent to the paretic leg(s) in order to provide movement assistance, and the other therapist standing behind the patient to shift body weight [6–9]. Hesse et al. showed that treadmill training with partial body weight support compared favorably to the then prevalent Bobath method in improving both gait ability and walking velocity in stroke patients [10]. The Bobath method attempts to restore more physiological gait pattern by applying tone-inhibiting exercises and motor tasks while patient is in a seated, standing or lying position [11]. BWSTT became the “gold standard” of gait rehabilitation with a meta-analysis of 21 randomized controlled trials (RCTs), suggesting that both gait speed and walking distance improved after BSWTT [12]. Increased brain activity has been observed during BWSTT in fNIRS [13] and after BWSTT in fMRI scans of stroke patients making ankle pointing movements, implying that the intervention went beyond adaptation and truly has neuro-recovery potential [14].

As clinicians began to fully incorporate the assumption that body weight supported treadmill training (BWSTT) delivered by 2 or 3 therapists per stroke patient was indeed “best practice,” automating BWSTT appeared to be the next logical step. Engineers developed robotic tools to replace this grueling, laborious, and ergonomically challenging approach. The devices can be classified into two main types: exoskeletons [15–17] and end-effector robots [18–21]. Both of these forms have shown promise in small pilot studies [22–26]. Yet when these robots were compared to usual care as practiced in the US in both sub-acute and chronic stroke populations, results failed to show the expected efficacy of the intervention [27, 28].

Furthermore, contrary to its clinical proponents, an NIH-sponsored randomized clinical trial demonstrated that the BWSTT did not lead to results superior to those from a much simpler and basic kitchen-and-sink home program that focused on only strength and balance training [29, 30]. This study, known as LEAPS, a 2011 Randomized Control Trial with over 400 stroke patients, highlighted that the goal of rehabilitation robotics cannot be to simply automate current rehabilitation practices. For the most part, automation lacks an evidential basis: a scientific basis is needed for the development of effective robotic therapy.

Knowledge of human sensorimotor control has matured to the point where a fundamental theory of walking is within reach. To enable the application of robotics to assist walking, we developed a competent model of human walking. By “competent model” we mean that it may only be a first approximation of a fundamental theory, but it is good enough to improve the design of robots and regimens for lower extremity therapy. In this working model, we considered walking in a wide range of realistic scenarios. Humans walk easily in diverse environments, including slopes, stairs, and uneven surfaces despite slow muscles, long neural communication delays and noise. Humans initiate and stop movements as well as change directions and transition between postures. Humans react to unexpected perturbations successfully. To accommodate real-life walking with all its variations, we propose a modeling approach that on a task-level encompasses discrete, rhythmic movements, and balance, which are decomposed into the dynamic primitives of submovements, oscillations, and mechanical impedances. An outline of this theory is presented in [31–36].

To enable the translation of this model to assist walking, we developed the MIT-Skywalker. The MIT-Skywalker is distinct from any of the existing rehabilitation robotic devices for gait [30, 37]. A key aspect of the design is the body weight support system. This paper presents some of the considerations and the results from a study of eight healthy subjects that guided us on the selection of the preferred support method. Here we will summarize our results on the displacement of the center of mass and the trunk under three conditions: subjects walking on the treadmill without any support, with 30% unloading supported by a harness hanging from an overhead system, and with a saddle/seat like support system.

### MIT-Skywalker

**Fig. 1:**
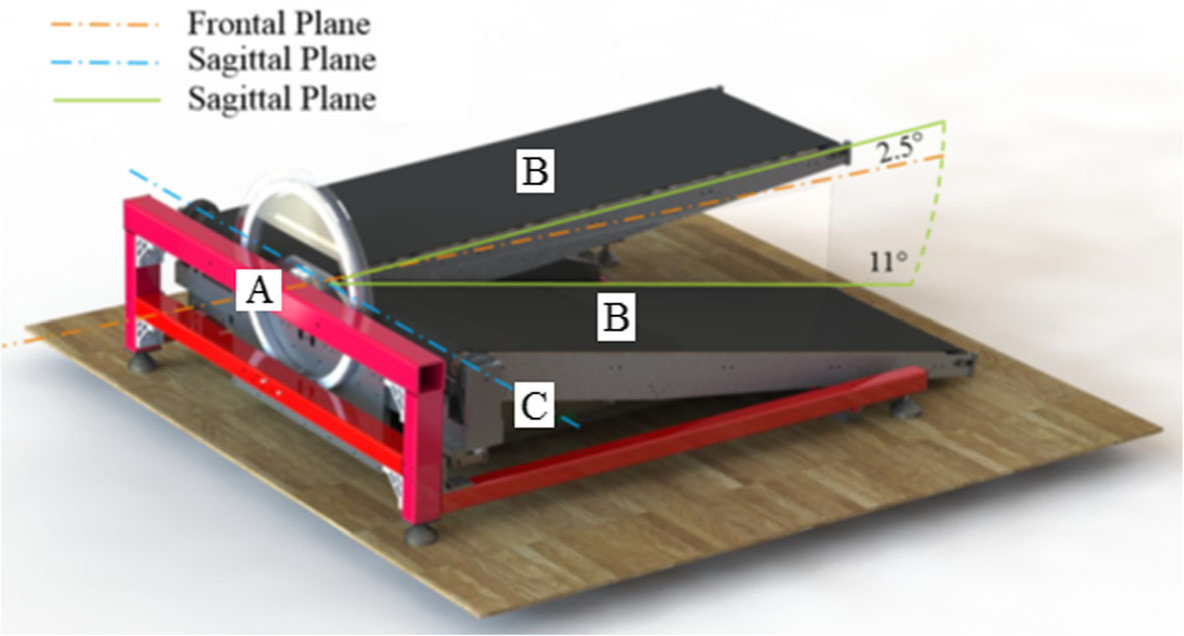
MIT-Skywalker

The MIT-Skywalker embodies the concept of passive walker in rehabilitation. The MIT-Skywalker walking system is shown in Fig. 1. The system has five active degrees of freedom: full system rotation in the frontal plane (a), independent treadmill actuation of both belts (b) and independent sagittal plane rotation of each treadmill about its front roller (c). Two of the drives are mirrored across the bisecting sagittal plane of the machine, resulting in three unique control systems drives [29].

**Fig. 2:**
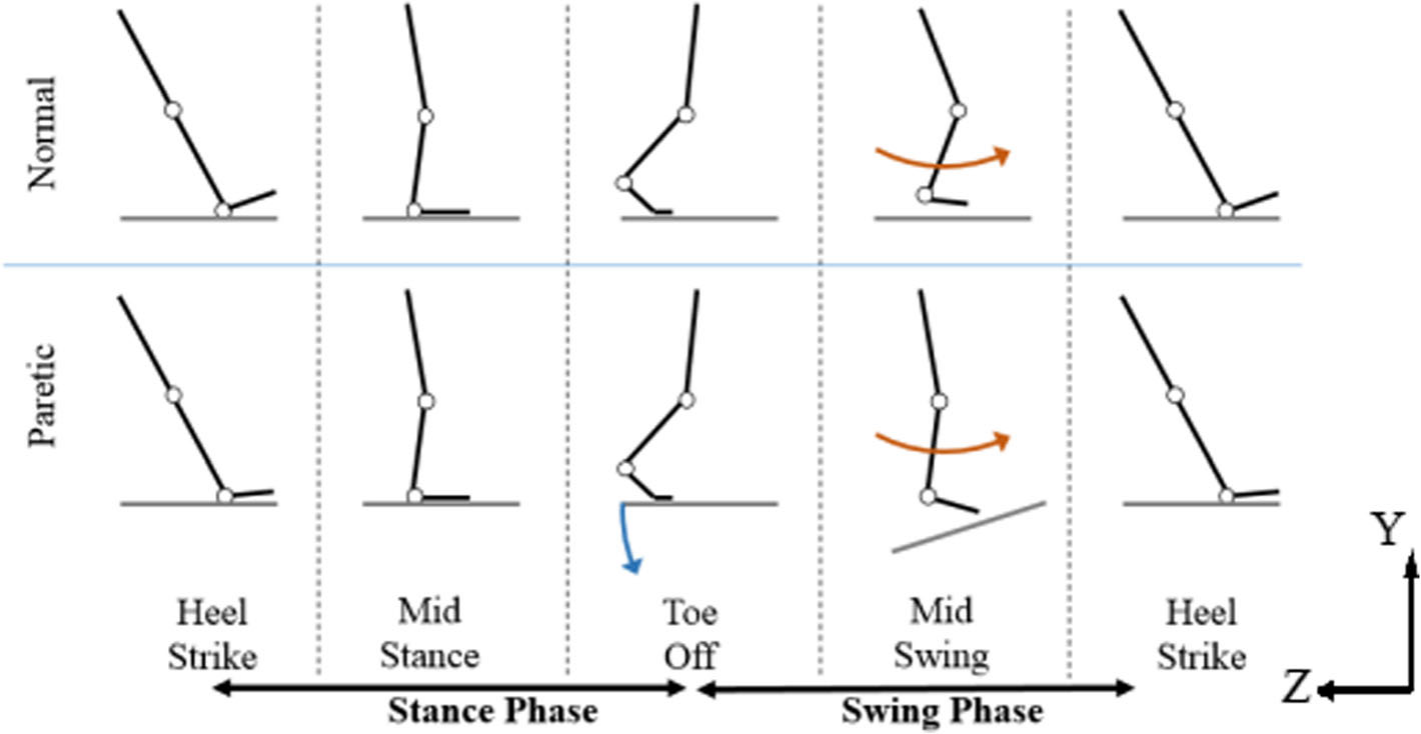
The MIT-Skywalker concept of assistance [26]

The MIT-Skywalker has a vision system to provide real time estimates of the angle of the thigh and shin so as to determine the posture and position of the patient (for additional details see [29, 30, 37, 38]). Figure 2 shows the principle of function of the MIT-Skywalker. In traditional BWSTT, Fig. 2(a), the leg supports the trunk while it moves backward relative to the trunk during the stance phase; at toe-off the support is shifted, the ankle completes a propulsive plantarflexion movement, and initiates a dorsiflexion movement to clear the ground initiating the swing phase. The walking surface is necessary during the stance phase, but it inhibits the leg during the swing phase and requires clearing the surface and propelling the leg forward. In the MIT-Skywalker, Fig. 2(b), the split treadmill moves the patient’s foot to the toe-off position. Once the vision acquisition system recognizes the heel *Z*-position has reached a minimum (patient-initiated swing phase), Fig. 2(b), the track is dropped, allowing the foot to swing forward freely for another step partially assisted by gravity (pendulum) and partially by patient’s effort.

### Body weight support system

In addition to the MIT-Skywalker moving platform, we considered two forms of actuated body weight support system: a harness hanging from an overhead system and a saddle/bicycle seat supporting from underneath.

**Fig. 3:**
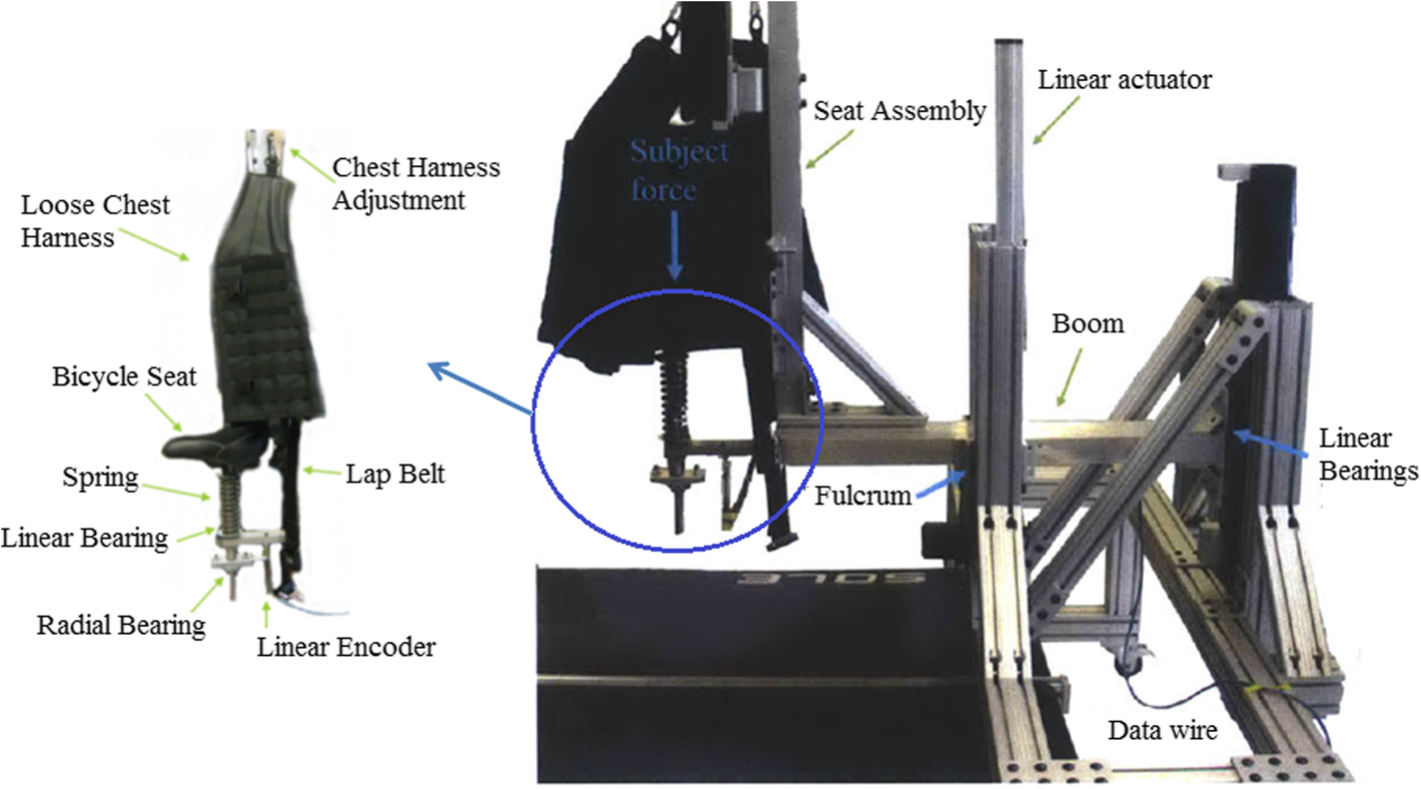
The MIT-Skywalker body weight support device

The harness system hanging from an overhead pontoon includes a commercial harness and the ability to unload and record the vertical displacement during training. The saddle/bicycle seat system includes a bicycle seat, a lap belt, and a loose fitting chest vest as shown in Fig. 3. The bicycle seat is mounted onto a shaft that is able to rotate in the transverse plane, but is restrained in other rotational DOFs by cylindrical linear bearings. A spring and linear potentiometer are employed to allow vertical displacement and to estimate the unloading and to record the vertical displacement during training. The system’s linear actuator sets the height of the BWS, thereby determining the percent of weight unloading. Note that the chest harness is used for safety and to “catch” the subject, thus preventing falls. The complete details of saddle/bicycle seat BWS can be found in [30, 37].

### On the use of Microsoft Kinect V2

**Fig. 4:**
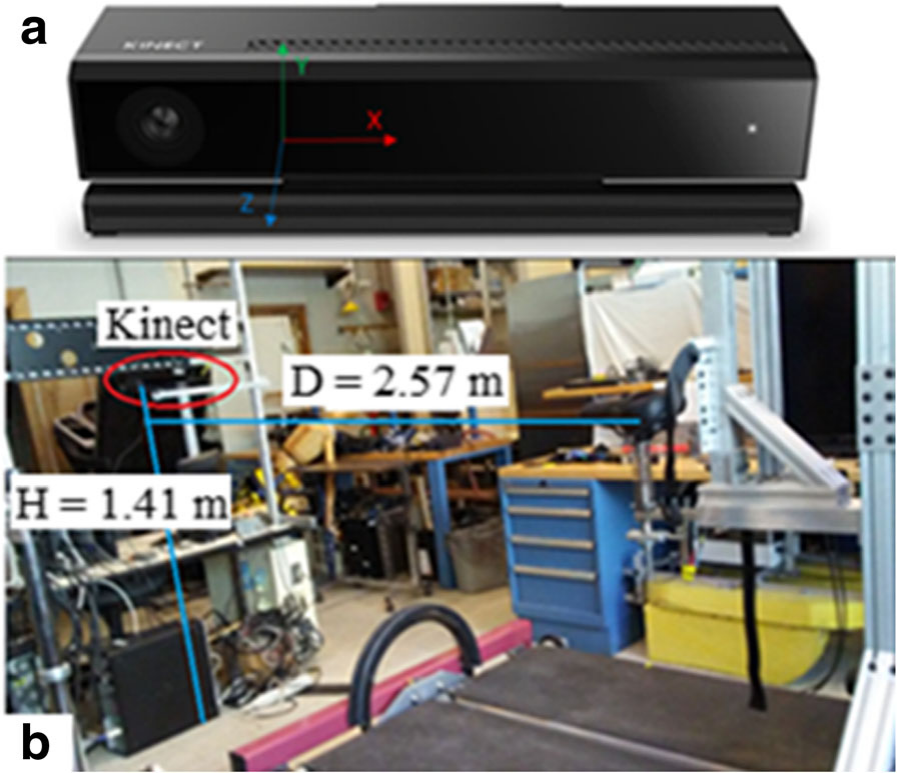
Kinect position and the reference system

The Microsoft^™^ Kinect® V2 (Kinect) is a low-cost markerless motion caption system designed for the Microsoft Xbox^™^. The Kinect has shown promise in clinical/biomechanics studies [39–47]. The version V2 was released in 2014 with improved depth and image hardware. It can track 3-D movement through its depth sensor and output the location of 25 joints in 3-D space at 30 Hz [48]. Previous studies [39, 40] showed the validity of the Kinect sensor to estimate the center of mass (COM) and we employed this approach in studying the different forms of body weight support. The COM location depends on body posture and on subject’s specific physical features. Typically, when the subject is standing upright (normal pose), the COM is located approximately at the center of the torso of the body [49]. Thus this paper uses the Kinect mid-spine joint as a coarse approximation of the COM during the gait cycle. Figure 4 shows the MIT-Skywalker and the placement of the Kinect [50]. The Kinect sensor was placed in front of the MIT-Skywalker and it recorded the 25 joint locations with software Kinect Studio v2.0. Figure 7(a) shows the 25 joints. The Kinect allows us to obtain the Cartesian position and the orientation of each joint. A complete joint description can be found in [45]. We used an open-source software [51] to extract/split the color and depth image as well as the skeleton data. Data was analyzed using the Matlab® 2015.

## Method

### Participants

Eight young adults [4 women and 4 males, age: 22.25 (±3.02) years, height: 1.68 (±0.10) m, mass: 67.4 (±11.91) kg] without any record of musculoskeletal or neurological disorder volunteered to participate. The study was approved by the MIT Committee on the Use of Humans as Experimental Subjects (COUHES).

### Experimental setup

**Fig. 5:**
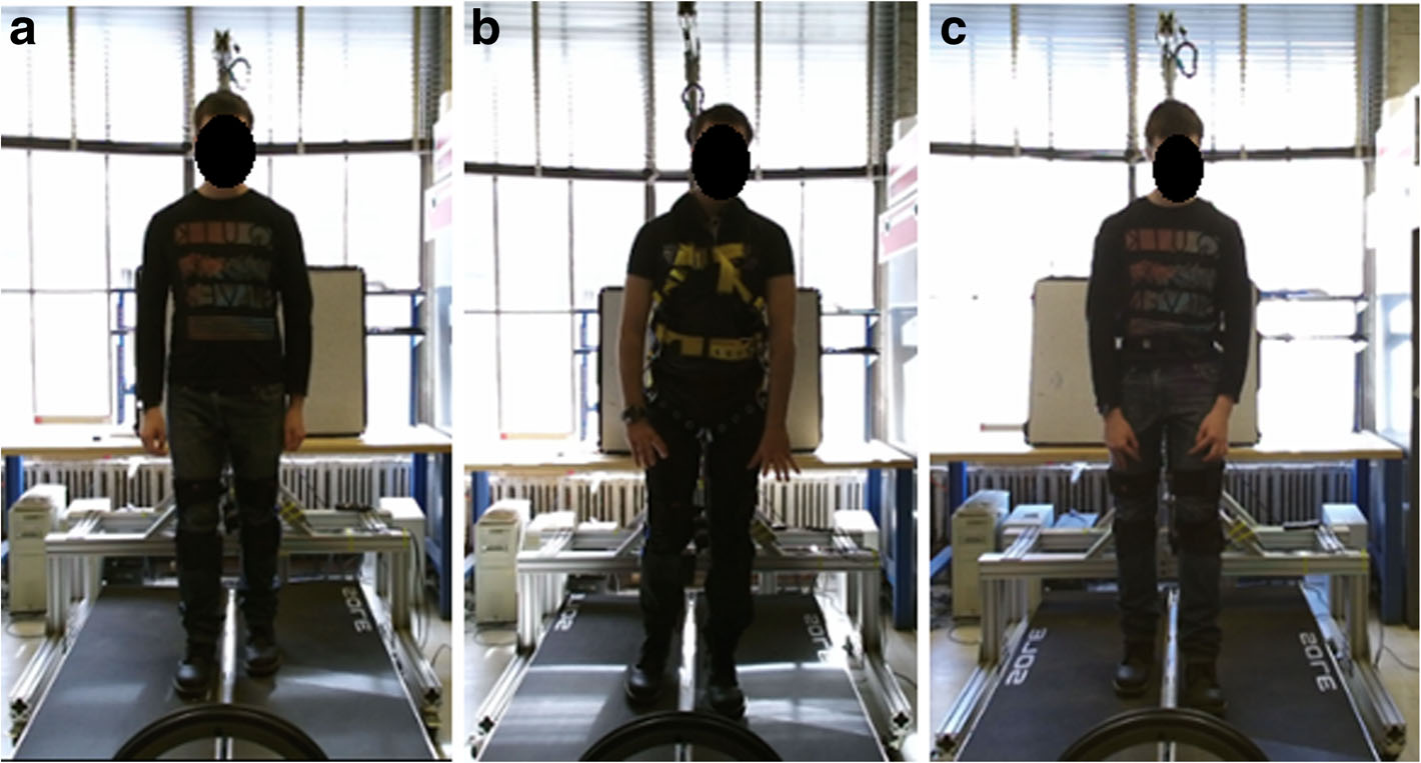
Testing apparatus on the MIT-Skywalker. (**a**) no BWS; (**b**) overhead harness; and (**c**) saddle/bicycle seat

**Fig. 6:**
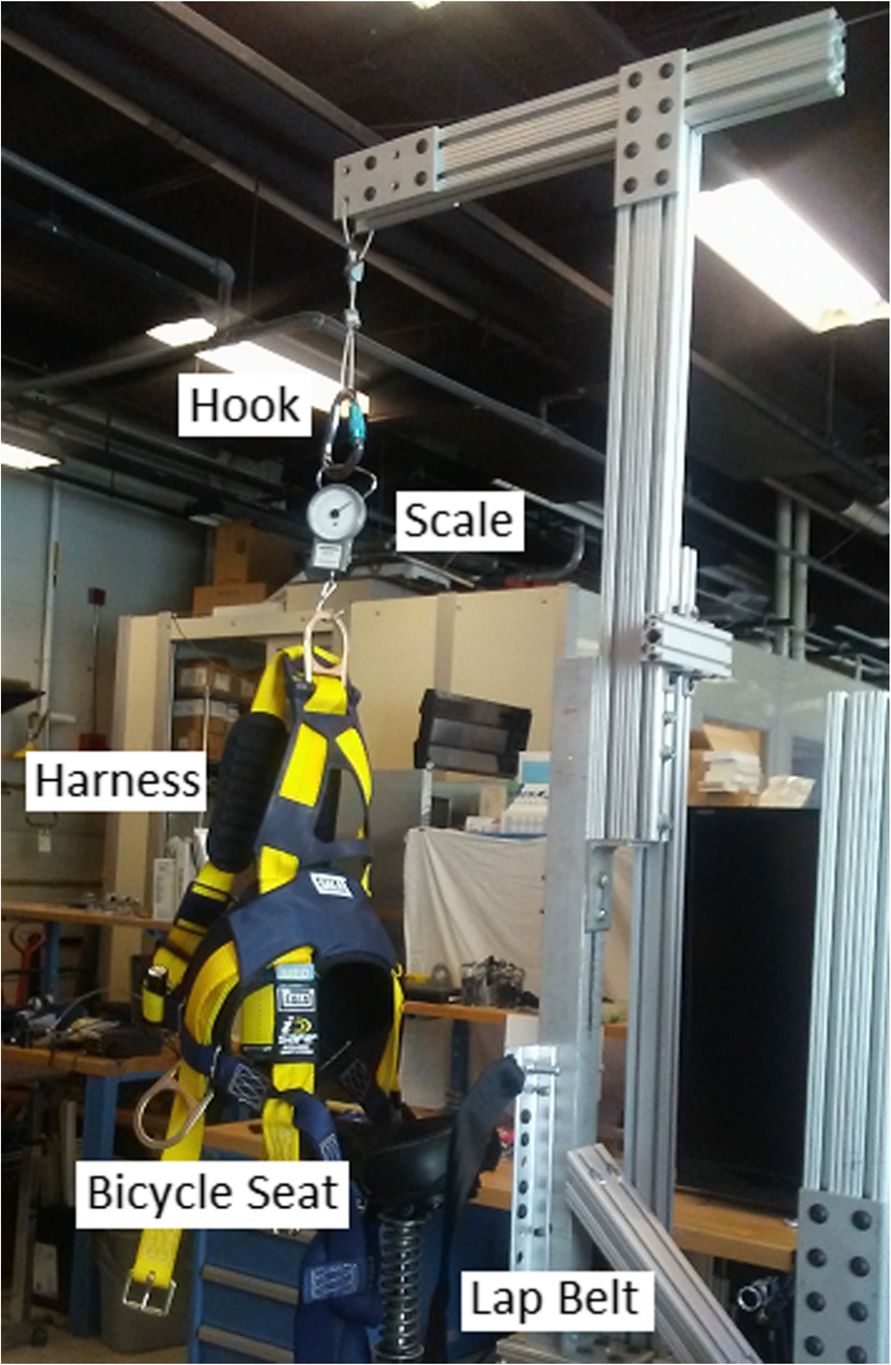
Experimental setup to compare different BWS

Prior to the trials, subjects were given a brief training/explanation session on the MIT-Skywalker to ensure they were acclimated to the device. The trial itself was organized as a 3 × 3 matrix. Three blocks were performed by the subjects walking on the MIT-Skywalker at three different walking speeds, corresponding to past experience with persons who had an impairment (v1 = 0.223 m/s, v2 = 0.447 m/s, and v3 = 0.671 m/s) and employing three support conditions: no BWS, overhead BWS, and the saddle/bicycle seat BWS (see Figs. 5 and 6). Each block lasted for 3 min. During the first minute subjects adapted to the selected speed. We acquired the data in the subsequent 2 min and the speed presentation was block-randomized. To check whether subjects were adapted after the familiarization session, we compared the amplitude of the mediolateral displacement at the beginning, middle and the end of the 2-min data acquisition period and tested for any differences in amplitude. For the two BWS conditions, the system was adjusted to unload 30% of the subject’s weight. The overhead BWS included a scale and a linear actuator (see Figs. 3 and 6).

**Fig. 7:**
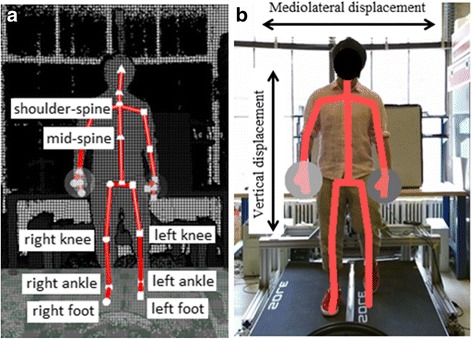
(**a**) The identified joints by the Kinect sensor; (**b**) color image with skeleton and the mediolateral and vertical displacement

For each block, we captured all the subject’s steps. For each step, we identified the maximum and minimum value of the mid- and shoulder-spine markers from the Kinect as shown in Fig. 7(a). We considered the mid-spine as an approximation of the human center of mass (COM). In addition, we considered the mediolateral and vertical displacement of the shoulder-spine marker [see Fig. 7(b)]. We utilized the color image to identify all steps. We excluded strides that lasted less than 10 frames.

### Statistical analysis

We compared the COM, as well as vertical and mediolateral displacements across different walking speeds and support using one-way ANOVAs and t-test. Before applying the ANOVA and the t-test, we checked for normality of the data thru a Jarque-Bera test. It confirmed the normality of the data to a significant level of 0.01. We employed ANOVAs to compare different speeds and support systems. The t-test was employed to compare two different speeds with the same support system. In these tests *pij* represents the *p-value* between the speed *i* and *j*. The significance level was set to 0.05. The asterisk in the Tables indicates statistical significance.

## Results

Tables 1, 2, 3 summarize the results. The coordinates *X* (mediolateral displacement) and *Y* (vertical displacement) represent the coordinates of mid-spine. The coordinates *X1* (mediolateral displacement) and *Y1* (vertical displacement) represent the coordinates of shoulder-spine. The mid-spine is identified as the COM and the shoulder-spine as the body trunk. There were statistically significant differences for the COM and mediolateral displacements in the “No BWS” condition with the exception of vertical body case *v1* vs *v2*. There were statistical differences for the COM and body mediolateral displacements for Overhead BWS in cases: *v2* vs *v3* and *v1* vs *v3*. Significant values were found in the cases *v1* vs *v2* (COM vertical displacements) and *v1* vs *v3* (COM vertical displacements and body vertical displacement). For the bicycle/saddle seat, the COM and body mediolateral displacements were statistically significant.

**Table 1:** No BWS condition. ** ANOVA and * t-test significance (*p* < 0.05)

No BWS	*v1*		*v2*		*v3*		t-test (*p*)		
COM range (cm)	mean	std	mean	Std	mean	std	*v1* vs *v2*	*v2* vs *v3*	*v1* vs *v3*
X**	11.11	2.01	9.11	1.50	7.70	1.71	0.0032*	0.0218*	3.2067e^-04*^
Y**	1.03	0.22	1.40	0.30	1.81	0.39	0.0063*	0.0032*	0.0015*
Body range (cm)	mean	std	mean	Std	mean	std	*v1* vs *v2*	*v2* vs *v3*	*v1* vs *v3*
X1**	12.64	2.50	10.19	1.85	8.33	1.97	0.0038*	0.0114*	5.1190e^-05*^
Y1**	1.11	0.26	1.41	0.30	1.80	0.39	0.0582	0.0079*	0.0052*

**Table 2:** Overhead BWS harness condition ** ANOVA and * t-test significance (p < 0.05)

No BWS	v1		v2		v3		t-test (*p*)		
COM range (cm)	mean	std	mean	Std	mean	std	*v1* vs *v2*	*v2* vs *v3*	*v1* vs *v3*
X	9.12	1.75	8.33	2.19	6.96	2.00	0.1358	0.0111*	0.0171*
Y	0.76	0.20	1.03	0.32	1.09	0.35	0.0384*	0.1580	0.0275*
Body range (cm)	mean	std	mean	Std	mean	std	*v1* vs *v2*	*v2* vs *v3*	*v1* vs *v3*
X1	9.36	2.02	8.75	2.67	7.32	2.41	0.2021	0.0152*	0.0204*
Y1	0.86	0.23	1.11	0.41	1.14	0.37	0.1164	0.6357	0.0479*

**Table 3:** Saddle/Bicycle seat BWS condition. ** ANOVA and * t-test significance (p < 0.05)

No BWS	v1		v2		v3		t-test (*p*)		
COM range (cm)	mean	std	mean	std	mean	std	*v1* vs *v2*	*v2* vs *v3*	*v1* vs *v3*
X**	3.77	0.86	2.97	0.90	2.22	0.46	0.0029*	0.0173*	0.0014*
Y	0.67	1.09	0.70	0.15	0.68	0.14	0.6189	0.7260	0.7872
Body range (cm)	mean	std	mean	std	mean	std	*v1* vs *v2*	*v2* vs *v3*	*v1* vs *v3*
X1**	5.93	1.57	4.74	1.56	3.59	0.72	0.0101*	0.0446*	0.0038*
Y1	0.82	0.18	0.78	0.15	0.81	0.21	0.6045	0.6485	0.9285

## Discussion

The aim of the current study is to evaluate two forms of body weight support systems for potential use in the MIT-Skywalker. As walking speed increases in healthy subjects walking without a BWS, the vertical displacement of COM displacement in frontal plane increases. This result is consistent with previous work comparing the effect of walking speed on COM displacement [52]. We and others observed similar behavior when employing the overhead harness system [53]. That is not case when employing the bicycle/saddle system which is mounted on a spring that limits vertical displacement.

Furthermore, as walking speed increased we observed a decrease of the mediolateral displacement for all cases. The *X*_*COM*_ displacement was large at slow speeds and decreased at faster walking speeds. For individuals with gait pathology, the increased *X*_*COM*_ displacement at slow speed may indicate additional balancing challenges and we speculate on whether one might gain additional benefits when training at low speeds (see Tables 1, 2, 3). To walk upright, bipeds need to activate different mechanisms of balance and these may be affected due to neurological injuries and aging [30, 54]. Balance training has been shown to reduce postural asymmetry associated with hemiparesis and was a part of the home-based protocol in the LEAPS study [31]. Furthermore, in the feasibility study of the MIT-Skywalker employing the bicycle/saddle support [30, 37], three patients with very different levels of impairment showed substantial improvement on the Berg Balance Test after a one-month study [30]. These initial results were very promising [30] and we plan to commence a larger set of clinical studies to determine whether our training approach with the MIT-Skywalker and its bicycle/saddle support leads to superior results as compared to usual care.

**Fig. 8:**
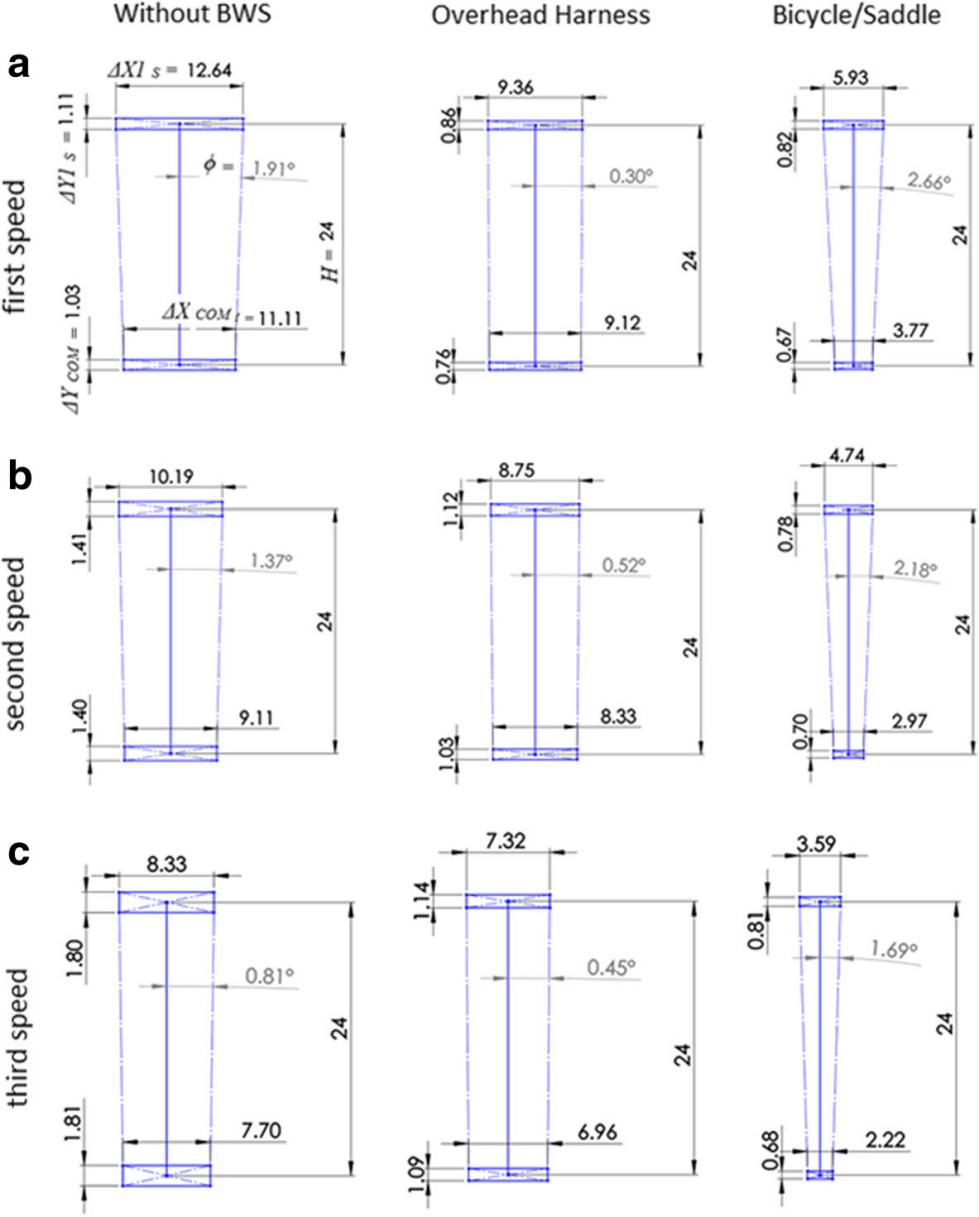
Comparison different BWS. Units in [cm]. (**a**) speed of 0.223(m/s); (**b**) speed of 0.447(m/s); and (**c**) speed of 0.671(m/s)

Figure 8 shows a graphical comparison of performance at three different speeds in different types of BWS. *ΔX*_*COM*_ and *ΔX1*_*S*_ represent the mediolateral range displacement for the mid-spine and shoulder-spine markers, *ΔY*_*COM*_ and *ΔY1*_*S*_ represent the vertical range displacement for the same joints, *H* represents the mean distance of the mid-spine and shoulder-spine for all subjects, and *ϕ* represents the inclination of the trunk.

Note that when using the overhead harness, the trunk has the smallest inclination at all the speeds, Fig. 8. Indeed, the overhead harness imposes a major constraint for trunk inclination. On the contrary, the bicycle/saddle system has the largest inclination which affords balance training. Furthermore, in [54, 55] it was shown that trunk exercises performed on an unstable surface improved the trunk muscle activation, postural control, and the gait speed in patients with stroke. There was further improvement observed in acute stroke involving trunk control and functional balance when using a dynamic surface (physio ball) compared to results from the same exercises being performed on a static surface (physio plinth) [56]. The authors suggested that dynamic practice leads to increased muscle activity with increased demands on postural control, voluntary trunk movements and an increased response to postural perturbations [56, 57].

In the reference [58], it was shown that trunk-functional assessment post-stroke is important as a requirement for predicting the probable clinical outcome of the patient with stroke and in designing an effective rehabilitation protocol. In the reference [59], 30 post stroke patients were divided into two groups. Group A (experimental group) received trunk rehabilitation, balance training and conventional physiotherapy; Group B (control group) received only conventional physiotherapy. The experimental group demonstrated additional improvements in the trunk impairment scale, Berg balance scale, gait speed, and cadence post intervention as compared to the control group. Swinnen et al. [60] analyzed the three dimensional movements of the trunk in healthy persons during walking on a treadmill with different levels of BWS and concluded that, compared with 0% BWS, the lateral flexion of the trunk on the pelvis decreased significantly at 10 to 50% BWS.

Individuals with gait disturbances due to limb loss, neuromuscular pathology, vestibular dysfunction, stroke, or aging elderly have reduced gait speed [57, 61]. As discussed earlier at slow gait speeds, there is an increase in the mediolateral COM motion which increases demand on balance traits [41]. In previous work with the MIT-Skywalker [29, 37], we tested the effect of the bicycle/saddle BWSS on a healthy subject up to 50% unloading. The bicycle/saddle system had a small effect on the kinematics, showing a slightly decreased knee angle. Without the seat, the swing phase initiates later. The use of the seat limited the hip angle [37]. In a recent study [59], we evaluated different shapes of seats to assess this limitation. Results with 10 healthy subjects demonstrated a reduction of 31% in hip flexion when using the seat. There was no statistically significant change in hip extension. To address this limitation, we recently tested an alternate bicycle/saddle seat and that restricted the hip flexion by only 19%. No statistical difference was found in the degree of pelvic rotation with the new seat (Gonçalves RS, et al.: MIT-Skywalker: evaluating comfort of bicycle/saddle seat, 15th IEEE international conference on rehabilitation robotics, unpublished).

Different research groups studied the influence of an overhead BWS on normal human gait [62–64]. Finch and colleagues found on 10 healthy subjects walking on a treadmill that the amplitude of movement of hip and knee decrease with the use of an overhead harness [62]. They speculated that the harness could limit the vertical displacement of the body, matching our results (see Fig. 8 smaller vertical displacement). Fischer showed that the use of the Biodex BWS had sizeable reductions in lower joint kinematics and kinetics in healthy subjects with increased unloading during over ground gait. The difference between the “no harness” and 30% unloading was 27.3% in the hip flexion [63]. Sousa demonstrated a decrease in the range of hip motion with 30% overhead BWS in stroke subjects during over ground walking [64]. One might suggest that an overhead BWS unfavorably influences balance training due to its pendulum-like behavior [30, 65, 66]. The use of the bicycle/saddle offsets the force just below the body’s center of mass, preserving the inverted pendulum behavior [30]. Furthermore, Kataoka and colleagues found on 6 healthy subjects walking on an instrumented treadmill (ITR5018, Bertec Corp, USA) that the ground reaction force profile employing a bicycle/saddle seat resembled the two peak profiles of “no harness” condition, while the overhead BWS profile was markedly distinct with a single peak [67]. In summary, a literature comparison on the impact of the overhead harness system and the bicycle/saddle on the hip joint kinematics showed a similar influence [29, 37, 62–64]. Of course there are more sophisticated smart versions of overhead BWS like Zero-G and FLOAT but those are not inexpensive [65, 68].

The bicycle/saddle system has additional advantages. In addition to affording realistic balance training as it supports the patient closer to the center of mass, the bicycle/saddle system allows notably faster don-on and don-off than the overhead harness system. Therefore, a patient receives longer actual training in a pre-set time period.

**Fig. 9:**
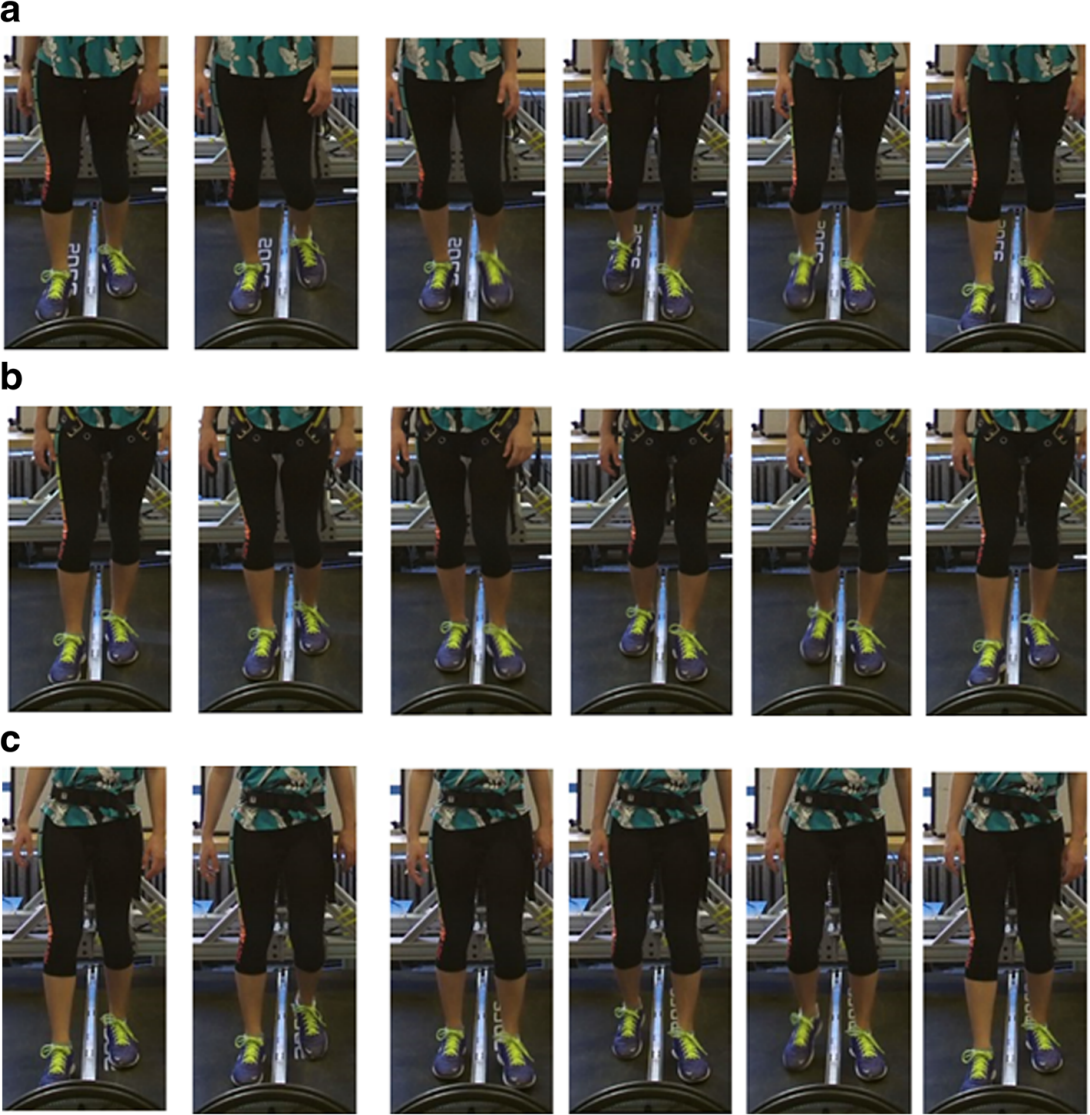
Compare different BWS. (**a**) Without BWS; (**b**) With Harness; (**c**) MIT-Skywalker

There is also a perceived safety advantage for the bicycle/saddle type BWSS. The MIT-Skywalker employs a split treadmill system which permits different tread speeds for each leg. The asymmetric speed programs focus on altering the step-length asymmetry via speed distortion (asymmetric split-belt speeds). A risk analysis includes the potential that a person using the overhead harness system might land a foot on the opposite tread which is undesirable. This is less of a concern on the bicycle/saddle system that centers the patient at all times [30, 37]. Figure 9 shows images of the three tested cases and one can note that subjects are centered all the time when employing the bicycle/saddle BWS. Subjects reported some level of discomfort but overall reported that it was more comfortable to walk on the bicycle/saddle BWS than the overhead BWS one.

Last but not least, the bicycle/saddle support system allows us to install the system in most clinics without major space alterations. In the US, regulations require a minimum ceiling height of 8 ft (2.4 m) and many devices, including many overhead BWS systems, require major space alterations even in code compliant facilities.

## Conclusion

In this paper we presented a comparison between two forms of BWS used by healthy subjects. We compared the “traditional” overhead harness with the bicycle/saddle system. Both cases presented different COM and shoulder-spine values when compared to the unsupported case. The bicycle/saddle system had the highest values for the mediolateral inclination and better centered the patient to the split treadmill, hence enhancing safety. The overhead harness BWS showed the smallest inclination for all the speeds, which might be adequate for rhythmic training but limits its potential for balance training. We believe our results may be transferable to any other devices employing simple BWS systems. We are presently 1) optimizing the bicycle/saddle seat shape to improve comfort, 2) developing a closed-loop control to adjust saddle support during training, and 3) planning clinical trials to assess whether our approach will lead to better results when compared to those produced using usual care.

## Abbreviations

BWSTT: Body weight supported treadmill training
COM: Center of mass
RCTs: Randomized controlled trials;
